# Effect of Ultraviolet Photofunctionalization on Implant Stability and Marginal Bone Loss in Early-Loaded Dental Implants: A Triple-Blind Randomized Split-Mouth Clinical Trial

**DOI:** 10.7759/cureus.117800

**Published:** 2026-10-12

**Authors:** Awab Midani, Issam Alkhouri

**Affiliations:** 1 Department of Oral and Maxillofacial Surgery, Damascus University Faculty of Dentistry, Damascus, SYR

**Keywords:** dental implants, early loading, implant stability, marginal bone loss, osseointegration, resonance frequency analysis, split-mouth randomized clinical trial, ultraviolet photofunctionalization

## Abstract

Background

Ultraviolet (UV) photofunctionalization has been proposed as a simple surface modification technique capable of restoring the biological activity of aged titanium implants and enhancing early osseointegration. Investigating the clinical performance of this technique under early loading protocols is essential to optimize patient outcomes. This randomized clinical trial evaluated the effect of UV photofunctionalization on implant stability and marginal bone loss (MBL) in early-loaded dental implants.

Methods

A triple-blind randomized split-mouth clinical trial was conducted involving 10 patients requiring bilateral posterior mandibular implant placement. Each participant received one UV-photofunctionalized implant and one untreated control implant. UV-C photofunctionalization was performed for 24 hours before surgery according to the manufacturer's protocol. Implant stability was assessed using resonance frequency analysis and expressed as the implant stability quotient (ISQ) immediately after implant placement (baseline), at three weeks, and at four weeks, when prosthetic loading. MBL was evaluated using standardized digital periapical radiographs obtained immediately after implant placement and one year after functional loading. Statistical analyses were performed using a linear mixed-effects model and paired statistical tests appropriate for the split-mouth design, with statistical significance set at P < 0.05.

Results

All 20 implants survived successfully and completed the one-year follow-up without biological or prosthetic complications. Baseline ISQ values were comparable between the UV and control groups (P = 0.694). At three weeks, the UV-treated implants demonstrated significantly higher ISQ values than the control implants (82.60 ± 2.12 vs. 76.70 ± 3.37; P < 0.001). This difference remained significant at four weeks (82.90 ± 2.28 vs. 75.40 ± 3.66; P = 0.005). The mean MBL after one year was lower in the UV group than in the control group (0.155 ± 0.230 mm vs. 0.209 ± 0.247 mm); however, the difference was not statistically significant (P = 0.607).

Conclusions

UV photofunctionalization significantly enhanced implant stability during the early healing period under an early loading protocol, suggesting accelerated early implant stabilization. Although MBL was numerically lower in the UV-treated implants, no significant long-term radiographic benefit was observed after one year. UV photofunctionalization may represent a simple and effective adjunctive strategy for improving early implant stability, particularly in clinical situations requiring early loading. Larger randomized clinical trials with longer follow-up are warranted to confirm these findings.

## Introduction

Dental implants have become a predictable and widely accepted treatment for replacing missing teeth because they provide favorable functional and esthetic outcomes with high long-term success rates. The long-term success of implant therapy depends primarily on achieving osseointegration, which is defined as a direct structural and functional connection between the implant surface and the surrounding living bone. Successful osseointegration provides the biological foundation for implant stability, which is essential for functional loading and long-term clinical success [1-3].

Implant stability is considered one of the most important determinants of successful osseointegration and long-term implant survival. It consists of primary stability, which is achieved through the mechanical engagement of the implant with the surrounding bone at placement, and secondary stability, which develops during healing as a result of bone remodeling and new bone formation around the implant. Several factors, including bone quality, implant design, surgical technique, and implant surface characteristics, can influence implant stability [4,5].

Early loading protocols have gained increasing acceptance as advances in implant design and surface modification have improved the predictability of implant treatment. Clinical evidence has shown that early loading can achieve favorable survival rates and maintain marginal bone stability when adequate primary stability is obtained and appropriate case selection is ensured. Nevertheless, achieving rapid and reliable osseointegration during the early healing period remains a prerequisite for the success of early-loaded implants [6].

In addition to implant stability, preservation of peri-implant marginal bone is a fundamental determinant of long-term implant success. Marginal bone loss (MBL) is a widely accepted radiographic outcome for evaluating implant performance and peri-implant tissue health, reflecting the biological response of the surrounding bone to functional loading [7,8]. According to the widely accepted success criteria, MBL should not exceed approximately 1.5 mm during the first year after functional loading and 0.2 mm annually thereafter [2].

However, achieving these objectives may be compromised by the biological aging of titanium implant surfaces. Although newly manufactured titanium exhibits high bioactivity, this gradually declines during storage because of progressive hydrocarbon contamination, loss of surface hydrophilicity, and reduced osteoconductivity. These time-dependent physicochemical changes may impair the biological response at the bone-implant interface and delay early implant stabilization [9,10].

To overcome these limitations, several implant surface modification strategies have been developed to enhance the biological performance of titanium implants. Among these approaches, ultraviolet (UV) photofunctionalization has emerged as a simple and promising chairside technique performed immediately before implant placement. UV photofunctionalization restores the bioactivity of aged titanium surfaces by removing accumulated hydrocarbon contaminants, increasing surface energy, and converting the implant surface from hydrophobic to superhydrophilic. These physicochemical changes enhance protein adsorption and promote the recruitment, attachment, proliferation, and differentiation of osteogenic cells, thereby accelerating new bone formation and improving bone-to-implant integration. In vitro and animal studies have consistently demonstrated enhanced osteoconductivity, increased bone-to-implant contact, and faster osseointegration following UV treatment, suggesting that photofunctionalization may improve implant performance, particularly in clinical situations where rapid osseointegration is desirable, such as early loading protocols [9-14].

Several clinical studies have evaluated the effect of UV photofunctionalization on implant stability and osseointegration. Most clinical studies have reported favorable outcomes, including accelerated implant stability development, improved bone-to-implant integration, and maintenance of marginal bone levels, particularly in compromised bone conditions and implants with low primary stability [15-18]. However, the available clinical evidence remains limited because of the relatively small sample sizes, heterogeneous study designs, variations in implant systems, loading protocols, UV treatment protocols, and follow-up periods. Consequently, the clinical effectiveness of UV photofunctionalization, particularly under early loading protocols, has not yet been fully established [9,15-18].

Despite the promising results reported in previous clinical studies [15-18], further well-designed randomized clinical trials are needed to strengthen the clinical evidence regarding the effect of UV photofunctionalization on implant stability and MBL under early loading protocols.

Therefore, the aim of the present triple-blind randomized split-mouth clinical trial was to evaluate the effect of UV photofunctionalization on implant stability and MBL in early-loaded dental implants. Firstly, it was hypothesized that UV photofunctionalization would significantly enhance implant stability (primary outcome) during the early healing period compared to the untreated control implants. Secondarily, it was hypothesized that the UV-treated implants would exhibit lower MBL (secondary outcome) one year after functional loading than the control group.

## Materials and methods

Study design and ethical considerations

This triple-blind randomized split-mouth clinical trial was conducted at the Department of Oral and Maxillofacial Surgery, Faculty of Dentistry, Damascus University, Damascus, Syria, between 2022 and 2026. The study was conducted in accordance with the research protocol approved by the Faculty of Dentistry, Damascus University, after ethical approval was granted by the Biomedical Research Ethics Committee (BMREC) of Damascus University (approval number: DN-200825-494). The trial was retrospectively registered at ClinicalTrials.gov (identifier: NCT07694388). Written informed consent was obtained from all participants before enrollment. A total of 10 patients requiring bilateral dental implant placement were included in the study. Following a split-mouth design, each participant received two dental implants, that is, one UV-photofunctionalized implant (test group) and one untreated implant (control group), allowing each participant to serve as their own control.

Inclusion and exclusion criteria

Participants were considered eligible if they met the following inclusion criteria: adults aged 18 years or older who were willing to participate in the study, provided written informed consent, and agreed to comply with the study protocol and follow-up schedule; had good general health with no systemic contraindications to dental implant placement; demonstrated satisfactory oral hygiene and oral health status based on clinical examination; required dental implant placement with at least six months elapsed since tooth extraction; and presented with sufficient bone volume to allow implant placement without the need for bone augmentation procedures.

Patients were excluded if they exhibited parafunctional habits, such as bruxism; were heavy smokers (>10 cigarettes/day); had a history of radiotherapy or chemotherapy; were receiving long-term medications known to affect bone metabolism, such as corticosteroids; were pregnant or breastfeeding; presented with insufficient interocclusal space for prosthetic rehabilitation; had metabolic or bone disorders affecting bone quality or density, including osteoporosis and hyperparathyroidism; or demonstrated inadequate primary implant stability, defined as an insertion torque of <35 Ncm and/or a mean implant stability quotient (ISQ) of <65, making them unsuitable for the planned early loading protocol. Additionally, all clinical sites with poor bone quality, specifically soft trabecular bone corresponding to Class D4 bone density according to Misch's classification, were explicitly excluded from the study.

Sample size calculation

The sample size was calculated using the G*Power software (version 3.1; Heinrich-Heine-Universität Düsseldorf, Düsseldorf, Germany). Based on a two-tailed paired-samples t-test with a significance level (α) of 0.05 and a statistical power of 95%, a minimum sample size of seven implants per group was determined necessary to detect a large projected effect size ($d_{z}$) of 1.65. To compensate for potential dropouts and enhance the robustness of the study, the sample size was increased to include a total of 20 implants, equally allocated between the test and control groups (10 implants per group).

Randomization and allocation concealment

Immediately before surgery, treatment allocation was performed by an independent investigator who was not involved in patient recruitment, surgical procedures, outcome assessment, or statistical analysis. Simple randomization using coin tosses was employed to determine both the treatment allocation (UV-photofunctionalized or control implant) and the implant site (right or left posterior mandibular site). The independent investigator was solely responsible for performing the UV photofunctionalization procedure according to the randomization results and preparing the allocated implant before surgery. Following completion of the implant osteotomy, the assigned implant was handed to the operating surgeon already mounted on the implant carrier for insertion. This procedure ensured complete allocation concealment, as the operating surgeon remained unaware of the treatment assignment throughout the surgical procedure.

Blinding

The study adopted a triple-blind design. The participants, the operating surgeon (who also served as the clinical outcome assessor performing all ISQ measurements), and the statistician were completely blinded to the treatment allocation throughout the study. An independent investigator, who was not involved in the surgical procedures, clinical outcome assessments, or statistical analysis, was solely responsible for randomization, allocation concealment, and performing the UV photofunctionalization procedure. Before statistical analysis, the treatment groups were coded as Group A and Group B to ensure the blinding of the statistician. The allocation code was disclosed only after the completion of data collection and statistical analysis.

Implant system and UV photofunctionalization

All implants used in the study were commercially available titanium implants (Point Implant System, PointNix, Seoul, South Korea). All implants were 10 mm in length. Implant diameters were selected according to the implant site: 3.5 mm for premolar sites and 4.0 mm for molar sites.

For the test group, the implants underwent UV photofunctionalization using a UV Activator UV-A200 device (PointNix, Seoul, South Korea). According to the manufacturer's recommended protocol, the implants were exposed to continuous UV-C irradiation for a full duration of 24 hours while remaining sterile in their original packaging. This 24-hour continuous photofunctionalization procedure was fully completed approximately 24-48 hours prior to the surgical implant placement to ensure uniform bioactivity restoration and stable surface activation before clinical loading. Control implants received no UV treatment and were placed according to the same surgical protocol. All implants used in this study were selected from the same manufacturer manufacturing lot to ensure baseline surface conformity.

Surgical procedure

All surgical procedures were performed under local anesthesia using 2% lidocaine with 1:80,000 epinephrine. A crestal incision was made, followed by the elevation of a full-thickness mucoperiosteal flap to expose the alveolar crest. Implant osteotomies were prepared according to the manufacturer's recommended drilling protocol under continuous sterile saline irrigation. The implants were inserted at the crestal bone level.

Immediately after implant placement, primary implant stability was assessed by measuring insertion torque and ISQ. An insertion torque of at least 35 Ncm and a mean ISQ value of ≥65 were required to satisfy the predefined stability criteria for the early loading protocol. Implant stability was evaluated using a Mega ISQ II device (Osstell AB, Gothenburg, Sweden) based on resonance frequency analysis (RFA) [19]. ISQ measurements were obtained from four directions (buccal, lingual, mesial, and distal), and the mean value was recorded for analysis.

Healing abutments were connected immediately after implant placement, and the surgical flaps were repositioned and sutured using 3-0 nylon sutures. All patients received standard postoperative medications, including antibiotics, analgesics, and anti-inflammatory therapy, together with routine postoperative instructions. Sutures were removed seven days after surgery (Figure 1A, 1B).

**Figure 1:**
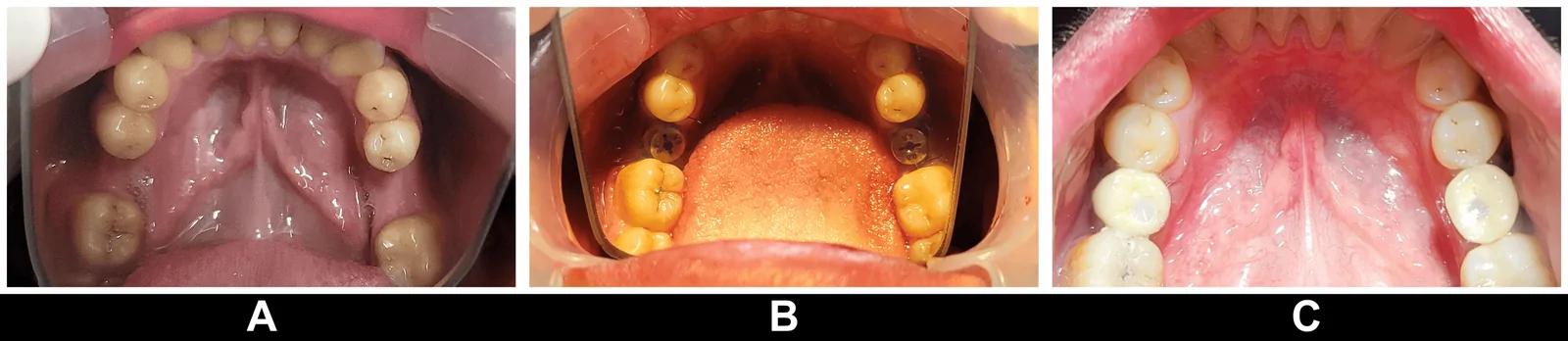
Representative clinical photographs showing the treatment phases. (A) Preoperative intraoral view of the bilateral posterior mandibular edentulous areas demonstrating clinical symmetry. (B) Immediate postoperative view showing the connected healing abutments (gingival formers) protecting the implant sites. (C) Definitive PFM fixed prostheses delivered at the four-week early loading protocol. PFM: porcelain-fused-to-metal

Prosthetic rehabilitation and early loading protocol

Three weeks after implant placement, implant stability was measured, and implant-level impressions were obtained using the open-tray impression technique. At four weeks after implant placement, definitive abutments were connected, and implant-supported porcelain-fused-to-metal (PFM) fixed prostheses were delivered according to the planned early loading protocol (Figure 1C). Occlusion was carefully adjusted using articulating paper to achieve stable centric contacts while eliminating premature and excessive occlusal contacts during excursive mandibular movements. Following prosthesis delivery, all patients were enrolled in the scheduled follow-up program according to the study protocol.

Outcome measures

The primary outcome of the study was implant stability, assessed using the ISQ. ISQ measurements were recorded immediately after implant placement (baseline), at three weeks, and at four weeks after implant placement immediately before prosthetic loading.

The secondary outcome was MBL. Standardized radiographic assessments were performed immediately after implant placement (baseline) and one year after prosthetic loading to evaluate changes in the marginal bone level around the implants.

Radiographic assessment

Standardized digital periapical radiographs were obtained using a digital sensor and the parallel technique assisted by a custom-made radiographic position-registering jig for each patient immediately after implant placement (baseline) and one year after prosthetic loading. The same radiographic technique, custom jig, and exposure parameters were used at both assessment time points (Figure 2).

**Figure 2:**
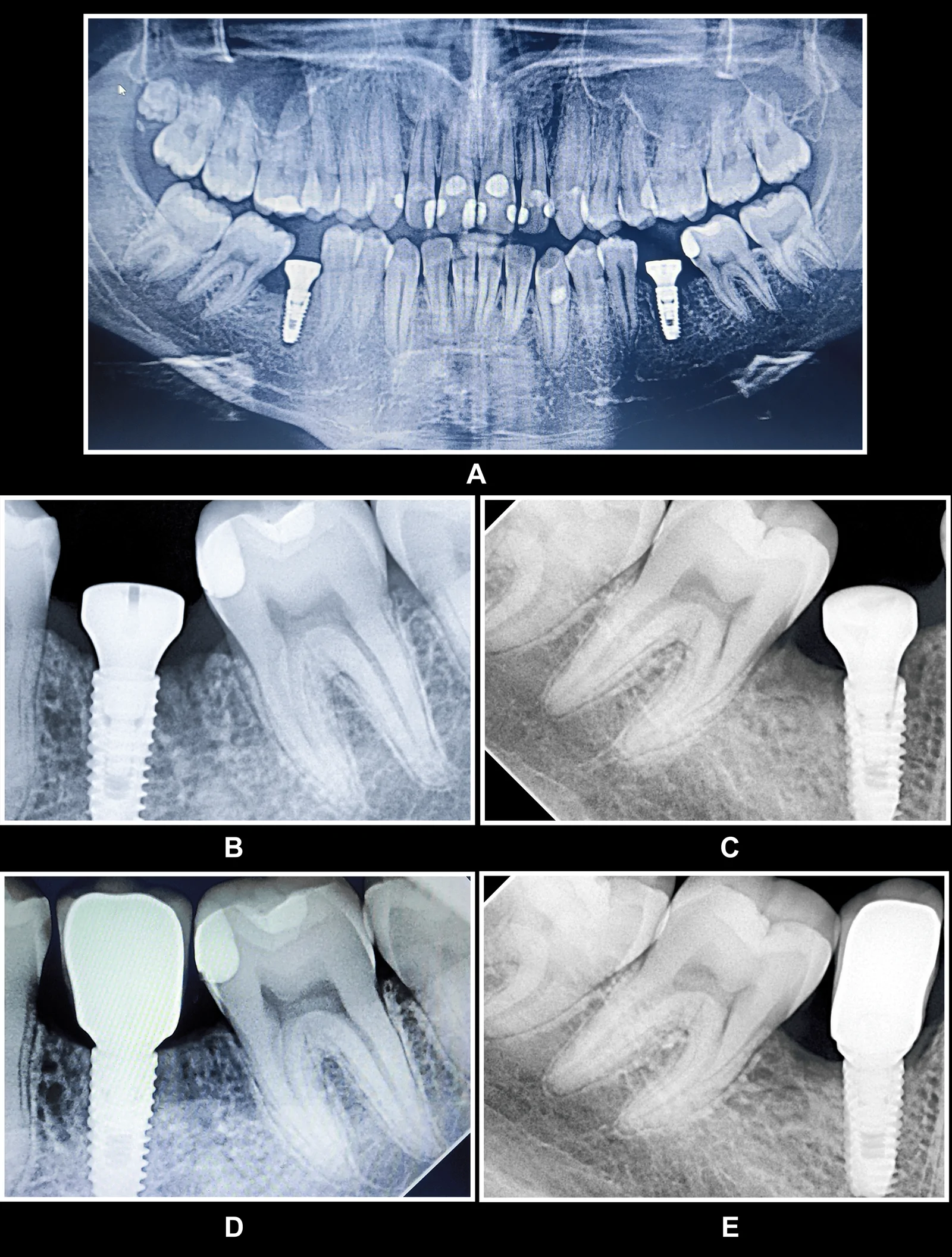
Radiographic evaluation at baseline and at the one-year follow-up. (A) Immediate postoperative panoramic radiograph showing the bilateral symmetric implant placement configuration within the posterior mandible. (B) Immediate postoperative digital periapical radiograph (baseline) of a UV-treated implant. (C) Immediate postoperative digital periapical radiograph (baseline) of a control implant. (D) One-year postoperative digital periapical radiograph of the UV-treated implant showing marginal bone-level outcomes. (E) One-year postoperative digital periapical radiograph of the control implant showing marginal bone-level outcomes.

Radiographic measurements were performed using the ImageJ software (National Institutes of Health, Bethesda, MD, USA). Each radiograph was calibrated using the known implant diameter to compensate for radiographic magnification.

The baseline radiograph obtained immediately after implant placement served as the reference image. Three reference landmarks were identified on each radiograph: (A) the first bone-to-implant contact on the mesial aspect, (B) the first bone-to-implant contact on the distal aspect, and (C) the implant apex. A reference line parallel to the implant platform was drawn through point C. The perpendicular distances from points A and B to this reference line were measured to determine the mesial and distal marginal bone levels, respectively. The same reference landmarks and measurement protocol were applied to the follow-up radiographs.

MBL was calculated for the mesial and distal aspects by subtracting the baseline measurements from the corresponding follow-up measurements. For each implant, the mean of the mesial and distal MBL values was used for statistical analysis. All radiographic measurements were performed independently by two blinded examiners, and the final value for each implant was calculated as the mean of the measurements obtained by both examiners. 

The participant flow throughout the study is illustrated in Figure 3.

**Figure 3:**
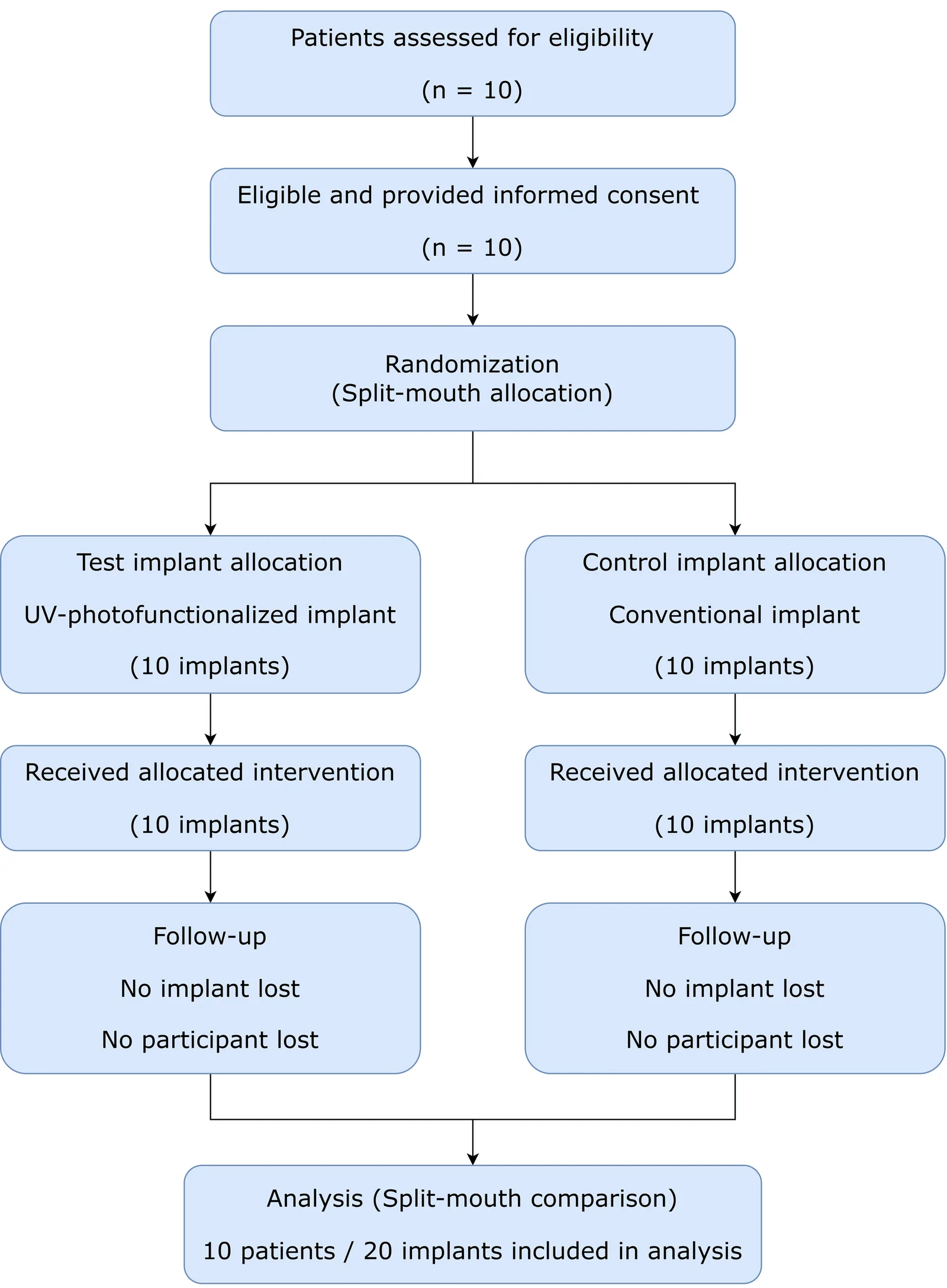
CONSORT flow diagram of patient enrollment, split-mouth randomization, allocation, follow-up, and analysis. All enrolled patients completed the one-year follow-up, and all implants were included in the final analysis. CONSORT: Consolidated Standards of Reporting Trials

Statistical analysis

Statistical analyses were performed using IBM SPSS Statistics for Windows, V. 25.0 (IBM Corp., Armonk, NY, USA). Data normality was assessed using the Kolmogorov-Smirnov test. Descriptive statistics were expressed as mean ± standard deviation (SD). Owing to the split-mouth study design and repeated measurements over time, a linear mixed-effects model was constructed to comprehensively evaluate changes in implant stability (ISQ values). The model incorporated treatment group (UV photofunctionalization vs. control), time (baseline, three weeks, and four weeks), and the treatment × time interaction as fixed effects, with the individual patient specified as a random effect to adjust for within-patient correlation. Sliced post hoc pairwise comparisons were performed using Bonferroni adjustment when appropriate. MBL, assessed one year after prosthetic loading, was compared between the two groups using the paired-samples t-test. A two-sided P-value of <0.05 was considered statistically significant.

To complement null hypothesis significance testing, effect sizes were calculated. Cohen's dz was utilized for parametric paired comparisons, and effect size r was calculated for the Wilcoxon signed-rank test. Effect sizes were interpreted as small (0.2), medium (0.5), and large (0.8). To ensure radiographic measurement reproducibility and eliminate potential inter-observer bias, MBL assessment via the ImageJ software was performed independently by two calibrated examiners, and the mean of the two independent measurements was utilized as the final dataset for statistical analysis.

The sequence and timing of the clinical procedures and outcome assessments are illustrated in Figure 4.

**Figure 4:**
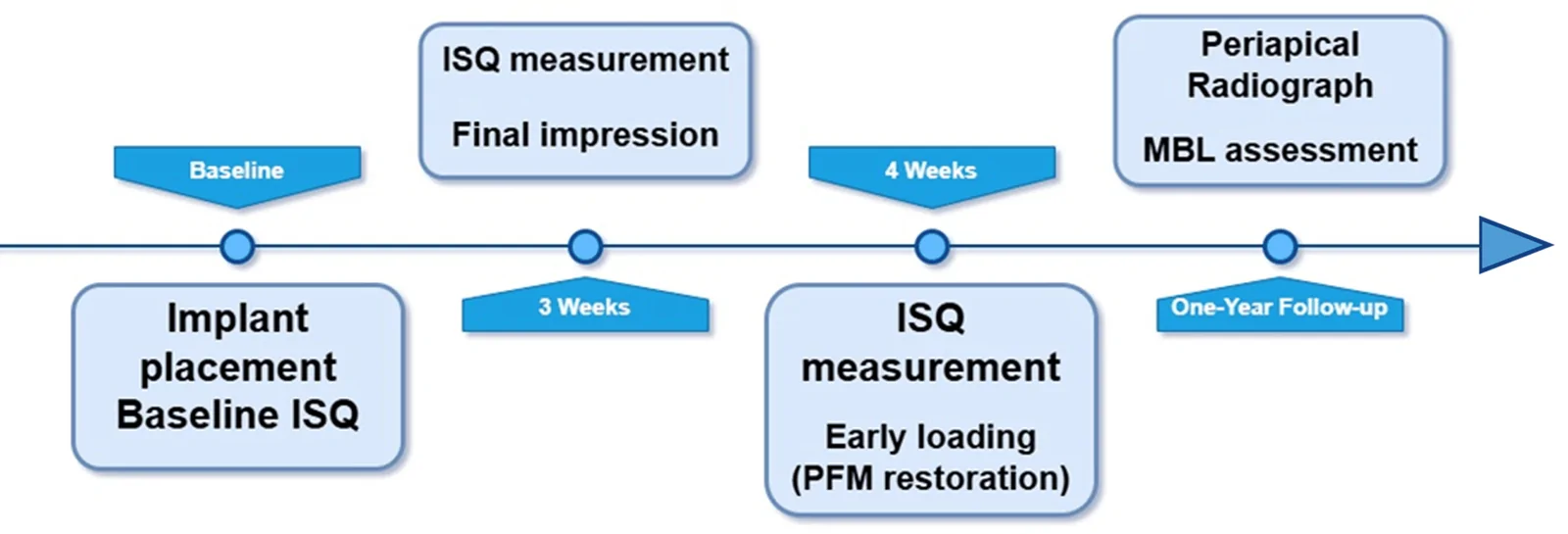
Study timeline showing the timing of implant placement, implant stability measurements, final impression, early prosthetic loading, and radiographic assessment of MBL. ISQ: implant stability quotient; PFM: porcelain-fused-to-metal; MBL: marginal bone loss

## Results

Participant characteristics

A total of 10 patients were enrolled in this study, and all completed the one-year follow-up. No participants were lost to follow-up. The study population consisted of six males and four females, with a mean age of 27.0 ± 11.8 years (range: 20-60 years). A total of 20 implants were placed, with 10 implants allocated to the UV photofunctionalization group and 10 to the control group according to the split-mouth design. All implants achieved the predefined stability criteria and were restored following the planned early loading protocol at four weeks after implant placement. No implant failures or biological or prosthetic complications were observed during the one-year follow-up period.

The baseline insertion torque was evaluated for all included implants to ensure adequacy for the early loading protocol. The data demonstrated a normal distribution (P > 0.05) by the Kolmogorov-Smirnov test. The mean insertion torque values were 37.50 ± 1.72 Ncm for the UV photofunctionalization group and 37.60 ± 1.84 Ncm for the control group. A paired-samples t-test demonstrated no statistically significant difference between the two groups (t = 0.14; P = 0.888), confirming equivalent baseline primary mechanical stability (Table 1).

**Table 1:** Comparison of baseline insertion torque between the UV photofunctionalization and control groups (n = 10 implants per group). UV: ultraviolet

Variable	UV group (mean ± SD) (Ncm)	Control group (mean ± SD) (Ncm)	Mean difference (Ncm)	t	P-value	95% CI for the difference			Effect size (Cohen's dz/r)	
Insertion torque	37.50 ± 1.72	37.60 ± 1.84	-0.1	0.14	0.888	(-1.66, 1.46)			-0.128 (negligible)	

Implant stability

Implant stability was assessed using the ISQ immediately after implant placement (baseline), at three weeks, and at four weeks before prosthetic loading. The ISQ values for both groups at each evaluation period are presented in Table 2.

**Table 2:** Comparison of implant stability quotient values between the UV photofunctionalization and control groups (n = 10 implants per group). UV: ultraviolet

Time point	UV group (mean ± SD)	Control group (mean ± SD)	Test statistic	P-value	Effect size (Cohen's dz/r)	
Baseline	80.30 ± 3.95	80.80 ± 2.82	t = -0.406	0.694	dz = -0.128 (negligible)	
3 weeks	82.60 ± 2.12	76.70 ± 3.37	t = 6.075	<0.001	dz = 1.921 (extremely large)	
4 weeks	82.90 ± 2.28	75.40 ± 3.66	z = -2.805	0.005	r = 0.887 (extremely large)	

The linear mixed-effects model revealed a highly significant main effect for time (P < 0.001) and a highly significant treatment × time interaction (P < 0.001), validating that the progression of secondary stability differed significantly between the groups over time. Sliced post hoc pairwise comparisons demonstrated that at baseline, there was no statistically significant difference in ISQ values between the UV photofunctionalization and control groups (80.30 ± 3.95 vs. 80.80 ± 2.82; P = 0.694), presenting a negligible effect size (Cohen's dz = -0.128). However, at three weeks, the UV photofunctionalization group showed significantly higher ISQ values than the control group (82.60 ± 2.12 vs. 76.70 ± 3.37; P < 0.001), demonstrating an extremely large effect size (Cohen's dz = 1.921). This statistically superior trend remained highly significant at four weeks before prosthetic loading (82.90 ± 2.28 vs. 75.40 ± 3.66; P < 0.005), with an extremely large effect size (r = 0.887), indicating accelerated early implant stabilization.

Within the UV photofunctionalization group, pairwise comparisons with Bonferroni adjustment demonstrated significant increases in ISQ values between baseline and three weeks (P = 0.028) and between baseline and four weeks (P = 0.037), whereas no significant difference was observed between the three- and four-week measurements (P = 0.837). In the control group, significant differences were observed between baseline and three weeks (P = 0.016) and between baseline and four weeks (P < 0.001), while the difference between the three- and four-week measurements was not statistically significant (P = 0.791).

MBL

MBL was evaluated one year after prosthetic loading. The mean MBL was 0.1547 ± 0.2301 mm in the UV photofunctionalization group and 0.2085 ± 0.2465 mm in the control group (Table 3). Although the UV photofunctionalization group exhibited a lower mean MBL, the difference between the two groups was not statistically significant (mean paired difference = −0.054 mm; 95% CI: -0.282, 0.175; paired-samples t = −0.533; P = 0.607), showing a negligible effect size (Cohen's dz = -0.169).

**Table 3:** Comparison of marginal bone loss between the UV photofunctionalization and control groups one year after prosthetic loading. UV: ultraviolet

Variable	UV group (mean ± SD) (mm)	Control group (mean ± SD) (mm)	Mean difference (mm)	t	P-value	Effect size (Cohen's dz)	
Marginal bone loss (mm)	0.1547 ± 0.2301	0.2085 ± 0.2465	−0.054	−0.533	0.607	-0.169 (negligible)	

## Discussion

The significant improvement in implant stability observed in the UV photofunctionalization group is consistent with the findings of several previous clinical studies. Suzuki et al. reported that UV-photofunctionalized implants exhibited accelerated implant stability development and faster osseointegration during the early healing period under immediate loading conditions [15]. Clinical reports by Funato and Ogawa [16] and Kitajima and Ogawa [17] further suggested that photofunctionalization is particularly beneficial in clinical situations requiring rapid establishment of implant stability, especially in compromised bone conditions or when primary stability is limited. Similarly, Choi et al. demonstrated enhanced early osseointegration and higher implant stability in UV-treated implants compared with UV-untreated implants [18].

The agreement between the present findings and previous clinical studies is biologically plausible and may be explained by the effects of UV photofunctionalization on titanium surfaces, which primarily enhance the early phase of osseointegration, during which implant stability develops most rapidly. Although these studies differed in loading protocols, implant systems, and patient characteristics, they consistently demonstrated improved early implant stability following UV photofunctionalization. UV irradiation has been shown to remove hydrocarbon contaminants that accumulate on titanium during storage, thereby restoring surface hydrophilicity and increasing surface energy, both of which improve the biological activity of the implant surface [10-12]. These surface modifications facilitate protein adsorption, osteoblast attachment, proliferation, and differentiation while promoting apatite deposition and increasing bone-to-implant contact during the early stages of healing [11-14]. Collectively, these biological responses accelerate new bone formation around the implant during the critical early healing phase, thereby promoting the more rapid development of secondary implant stability and explaining the higher ISQ values observed in the UV-treated implants [5,11-14].

Furthermore, a recent systematic review and meta-analysis concluded that photofunctionalization enhances the rate of implant stability development and may accelerate osseointegration, although the available evidence regarding its effect on MBL remains inconclusive [20]. Similarly, a recent narrative review emphasized that the clinical benefits of photofunctionalization are primarily associated with improvements in early biological responses at the implant surface, which may contribute to greater implant stability, particularly during the initial healing phase [21].

It is worth noting that because both the UV-treated and control implants successfully exceeded the clinical loading threshold (ISQ ≥65) by the fourth week, both groups were already clinically eligible for functional loading. Therefore, this 6-to-7-unit difference in ISQ values does not alter the absolute, binary clinical decision to proceed with the early loading protocol. However, the statistically superior stabilization observed in the UV group provides a significantly enhanced biomechanical safety margin, a faster stability turnaround, and greater structural predictability during the critical early healing phase, which is highly advantageous for clinical success.

Although the UV-treated implants demonstrated lower MBL than the control implants after one year, the difference was not statistically significant. This finding is consistent with the recent systematic review and meta-analysis by Lang et al. [20], which concluded that, despite the favorable effects of photofunctionalization on implant stability and early osseointegration, the available clinical evidence does not demonstrate a significant reduction in MBL. One possible explanation for the absence of a statistically significant difference is that marginal bone remodeling is influenced by multiple biological and mechanical factors, including surgical technique, prosthetic loading, peri-implant soft tissue health, oral hygiene, and host-related factors, which may reduce the measurable contribution of surface photofunctionalization over longer follow-up periods [6,20,21]. Nevertheless, the numerically lower MBL observed in the present study may indicate a favorable trend toward reduced MBL following UV photofunctionalization. Therefore, further randomized clinical trials with larger sample sizes and longer follow-up periods are required to clarify the long-term effect of photofunctionalization on peri-implant bone preservation.

Strengths of the study

The present study has several strengths. First, it was designed as a triple-blind randomized split-mouth clinical trial, minimizing selection, performance, and assessment bias while allowing each patient to serve as their own control. Second, the use of the split-mouth design reduced the influence of patient-related variables, such as bone quality, systemic conditions, and healing capacity. Third, implant stability was assessed using RFA at standardized time points during the early healing period, providing objective and reproducible measurements. In addition, all implants were placed using the same implant system and surgical protocol and were restored according to a standardized early loading protocol, thereby reducing methodological variability.

Study limitations

The present study has some limitations that should be acknowledged. First, the relatively small sample size may limit the generalizability of the findings and renders the MBL comparisons statistically underpowered to detect smaller clinical differences despite the lower numerical values observed in the UV group, and larger multicenter randomized controlled trials are needed to validate these results. Second, osseointegration was assessed using non-invasive clinical and radiographic parameters (ISQ and MBL) without direct histological or micro-computed tomography evaluation of the bone-to-implant contact. Third, the trial was registered retrospectively at ClinicalTrials.gov (identifier: NCT07694388) on July 3, 2026, which represents a limitation in strict programmatic adherence to international prospective clinical trial registry guidelines, although strict allocation concealment and comprehensive triple-blinding protocols were maintained throughout the trial to eliminate performance and assessment biases and all studied outcomes remained entirely unchanged following data collection. Finally, the study cohort consisted primarily of young, healthy, non-smoking individuals with a mean age of 27.0 ± 11.8 years who lacked metabolic bone disorders; while this cohort effectively minimized confounding host factors, it limits the immediate generalizability of our findings to older demographics or medically compromised patients, who are often the primary candidates for UV photofunctionalization. Furthermore, while a one-year follow-up period after functional loading is sufficient to evaluate early implant success and marginal bone remodeling trends, longer long-term follow-up periods are warranted to fully determine the definitive clinical and radiographic benefits of UV photofunctionalization under early loading protocols.

## Conclusions

Within the limitations of the present study, UV photofunctionalization significantly improved implant stability during the early healing period under an early loading protocol. However, no significant difference was observed in MBL after one year of functional loading. These findings suggest that UV photofunctionalization may enhance early implant stabilization and contribute to achieving favorable clinical outcomes during the early phase of treatment. Further randomized clinical trials with larger sample sizes, longer follow-up periods, and standardized photofunctionalization protocols are needed to confirm these findings and clarify the long-term clinical benefits of this surface modification technique.
